# MicroRNA-based strategies to overcome the immunotherapy barrier in pancreatic ductal adenocarcinoma

**DOI:** 10.3389/fimmu.2026.1744566

**Published:** 2026-04-28

**Authors:** Huiwen Xu, Qi Wang, Rubin Cao, Zhenpeng Qiu, Yuhui Liu, Junjie Hu, Guohua Zheng, Chen Cheng

**Affiliations:** 1 School of Pharmacy, Hubei University of Chinese Medicine, Wuhan, China; 2 Hubei Shizhen Laboratory, Wuhan, China; 3 Center of Traditional Chinese Medicine Modernization for Liver Diseases, Hubei University of Chinese Medicine, Wuhan, China; 4 Wuhan Jiulong Pharmaceutical Co. Ltd, Wuhan, China; 5 Division of Hepato-Pancreato-Biliary Surgery, Tongji Hospital, Tongji Medical College, Huazhong University of Science & Technology, Wuhan, China; 6 Key Laboratory of Chinese Medicine Resource and Compound Prescription, Ministry of Education, Hubei University of Chinese Medicine, Wuhan, China

**Keywords:** drug delivery, immunotherapy, microRNAs, pancreatic ductal adenocarcinoma, tumor microenvironment

## Abstract

Pancreatic ductal adenocarcinoma (PDAC) is a highly lethal malignancy that continues to pose a major clinical challenge, primarily due to the difficulty of early detection and the limited efficacy of existing therapeutic approaches. Immunotherapy, which has revolutionized the treatment of many other cancers, has shown limited success in PDAC, largely because of the complex and immunosuppressive features of its tumor microenvironment (TME). Consequently, strategies aimed at remodeling or modulating the TME have emerged as promising avenues for enhancing the therapeutical potential of immunotherapy in PDAC. MicroRNAs (miRNAs), a class of small non-coding RNAs, have emerged as key regulators of gene expression with multi-target capabilities and relatively low toxicity. Increasing evidence demonstrates that miRNAs play critical roles in regulating immune responses and shaping the TME across diverse tumor types, highlighting their considerable potential in improving immunotherapeutic outcomes in PDAC. In this review, we summarize the functional roles of miRNAs in PDAC and discuss the advantages of miRNA-based therapeutics compared with conventional treatments. We further examine current immunotherapeutic strategies for PDAC and highlight how miRNAs regulate immune activity and TME dynamics, providing mechanistic insights into miRNA-mediated immunotherapy. Finally, we discuss the major challenges limiting clinical translation, including off-target effects, toxicity, and delivery barriers and outline emerging delivery platforms that may enhance therapeutic efficacy. Besides, we explore how emerging technologies, such as artificial intelligence (AI), miniature soft robotics, and advanced 3D imaging ecosystems can be integrated into miRNA-based therapeutic strategies. Together, these innovations may pave the way for more effective, personalized, and patient-centered miRNA-based immunotherapies for PDAC.

## 1 Introduction

Pancreatic cancer (PC) remains one of the most aggressive and devastating malignancies, characterized by complex pathogenesis involving genetic susceptibility, chronic pancreatitis, and mutations in signaling pathway genes (1). Epidemiological research indicated that PC caused an estimated 50,550 deaths out of 64,050 new cases in the United States in 2023 (2). The latest data released by the National Cancer Center of the Chinese Academy of Medical Sciences reported 134,374 new cases and 131,203 deaths in 2022 (3). According to WHO statistics, PC currently ranks 12th in incidence and 6th in mortality, with a dismal five-year survival rate of only 13% (2). It is projected that by 2030, PC will become the second leading cause of cancer-related deaths worldwide (4).

Several modifiable risk factors contribute to the development of PC, including living habits, obesity, and related metabolic diseases. For instance, smoking, heavy alcohol consumption, and certain dietary patterns have been associated with a 2-3-fold increased risk (4). Overweight or obese individuals have an increased likelihood of developing PC (5). Histologically, pancreatic ductal adenocarcinoma (PDAC) accounts for approximately 90% of all PC cases, with other subtypes including acinar carcinoma, pancreatoblastoma, and neuroendocrine tumors. The majority of PC originate from precursor lesions known as pancreatic intraepithelial neoplasia (PanIN), which undergo stepwise genetic alterations, eventually developing into PDAC.

Only a small proportion of PDAC patients are eligible for surgical resection at diagnosis, making chemotherapy and radiotherapy become the primary treatment options for most cases (6). While the chemotherapy and radiotherapy existed some disadvantages, such as efficacy of treatment, chemoresistance, tumor microenvironment (TME) and safety profile, which make them great challenge for clinical PC treatment. Nowadays, immunotherapy has achieved notable success in various other cancers. Although several immunotherapies have been adapted to clinical PDAC treatment with great efficiency and unnotable side effects, PDAC poses a unique challenge due to its highly immunosuppressive TME. This TME is characterized by a dense extracellular matrix, tumor-associated macrophages (TAMs), myeloid-derived suppressor cells (MDSCs), and regulatory T cells, which together foster an immunosuppressive cytokine milieu—dominated by TGF-β, IL-10, and arginase-1—that inhibits T-cell activation. Additionally, PDAC exhibits significant genomic and antigenic heterogeneity, enabling the rapid outgrowth of tumor clones that downregulate target antigens, thereby driving immune escape and limiting durable treatment control (7–12). As a result, PDAC has shown considerable resistance to many immunotherapeutic strategies. So it will make sense to find the way to solve the obstacle of TME to make the immunotherapy more efficient.

MiRNAs, as small non-coding RNA molecules, have increasingly been recognized for their role in regulating multiple targets with low toxicity and modulating immune responses and the TME across multiple tumor types. In this review, we summarize the functions of miRNAs in pancreatic cancer and the advantages of miRNA-based therapy compared with traditional therapy. We conclude immunotherapeutic approaches for PC and examine the regulatory roles of miRNAs in shaping immune activity and the TME, offering new perspectives on potential mechanisms for miRNA-based immunotherapy. However, the clinical translation of miRNA-based therapy faces great obstacle due to toxicity, off-targeted effects, effective delivery systems are crucial for the clinical translation of miRNA-based therapeutics. We therefore outline key delivery strategies and highlight the most promising platforms for enhancing treatment efficacy. Together, these developments lay the groundwork for mechanism-driven innovations and improved delivery platforms, advancing the potential of miRNA-based immunotherapy in pancreatic cancer.

## 2.The current clinical landscape of PDAC

**Figure 1:**
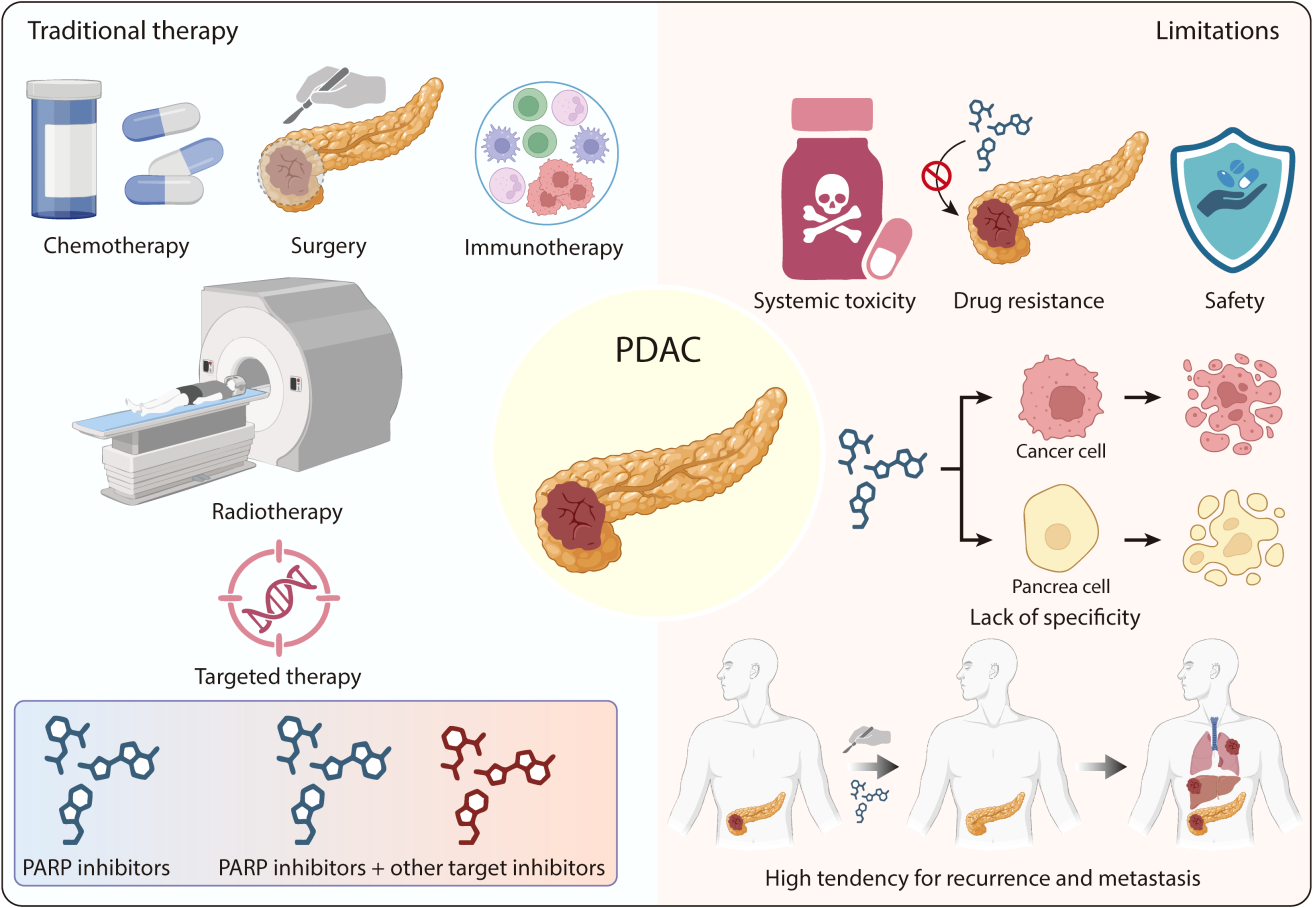
The traditional therapy of PDAC and limitations. The five primary treatment modalities include surgery, chemotherapy, radiotherapy, targeted therapy (e.g., PARP inhibitors, alone or in combination), and immunotherapy. Key limitations of these therapies include systemic toxicity, development of drug resistance, lack of tumor specificity, high tendency for recurrence and metastasis, and safety issues.

PDAC remains one of the most lethal malignancies, necessitating comprehensive therapeutic approaches to improve patient survival outcomes. Surgical resection is only eligible for 15% to 20% of patients who are diagnosed with localized pancreatic cancer (13). Other treatment modalities, including chemotherapy, radiotherapy, targeted therapy and immunotherapy have been employed to treat PDAC (**Figure 1**).

### 2.1 Chemotherapy

Chemotherapy remains a cornerstone in the management of PDAC, playing a pivotal role across adjuvant, neoadjuvant and advanced disease settings (14).

The primary therapeutic goal of the adjuvant chemotherapy is the eradication of microscopic residual disease following surgical resection to prevent recurrence FOLFIRINOX and gemcitabine (GEM)-based combination are chemotherapy options that have shown promising results. However, the treatment paradigm has shifted to modified FOLFIRINOX (mFOLFIRINOX) following the landmark trail, which extended median overall survival (OS) to 54.4 months compared with 35.0 months with GEM (p = 0.003). Subgroup analyses consistently favored mFOLFIRINOX over GEM. Nevertheless, due to the regimen’s considerable toxicity, over half of the patients required granulocyte colony−stimulating factor (G−CSF) support, and a significant proportion needed dose adjustments to maintain treatment tolerability (15).

Neoadjuvant chemotherapy aims to downstage tumors and facilitate surgical resection. Recent years have witnessed substantial progress in neoadjuvant chemotherapy for PDAC. Among them, studies on neoadjuvant chemoradiotherapy (CRT) have yielded promising results. In the PREOPANC−1 study, 246 patients were randomized to either upfront surgery followed by adjuvant GEM or CRT with GEM followed by surgery and adjuvant GEM. Long−term follow−up revealed a prolonged median overall survival in the neoadjuvant group (15.7 vs. 14.3 months), with 5−year OS rates of 20.5% and 6.5%, respectively. Subgroup analysis further indicated a significant survival benefit from neoadjuvant CRT (17.6 vs. 13.2 months, p = 0.029) (16).

Chemotherapy for advanced PDAC refers to palliative systemic chemotherapy aimed at prolonging survival and controlling disease progression rather than achieving cure. In advanced PDAC, NALIRIFOX and FOLFIRINOX should be the preferred options for patients who can tolerate these regimens, with GEM plus nab-paclitaxel remaining a viable alternative, particularly in patients unfit for triplet therapy (17). Among these, the PRODIGE trial demonstrated the marked advantage of FOLFIRINOX over GEM monotherapy in advanced PDAC, with median OS of 11.1 months and median progression-free survival (PFS) of 6.4 months in the FOLFIRINOX arm, compared to 6.8 and 3.3 months in the GEM arm, respectively. However, it should be noted that the FOLFIRINOX regimen is associated with increased toxicity and reduced quality of life, which limits its broad clinical application (18). Concurrently, the NAPOLI−3 trial provided compelling evidence supporting NALIRIFOX as a first−line therapy for advanced PDAC, showing a median OS of 11.1 months in the NALIRIFOX arm versus 9.2 months with GEM plus nab−paclitaxel (p = 0.036) (19).

Although chemotherapy remains the conventional and effective treatment approach, there existed some disadvantages such as limitation of efficacy in various subtypes of PDAC, the safety dosage of combined chemotherapy, chemoresistance and the obstacle of TME. Consequently, many other therapies were developed to conquer the difficulties or supplement the shortcomings of chemotherapy.

### 2.2 Targeted therapy

Emerging evidence underscores that chemotherapy efficacy in PDAC is influenced by molecular heterogeneity. For instance, the COMPASS trial demonstrated that patients with basal-like tumors exhibited significantly improved PFS when treated with FOLFIRINOX compared to those with classical subtypes (60% vs. 15%, p = 0.0002) (20, 21). Additionally, retrospective analyses indicate that patients harboring BRCA1/2 mutations exhibit significantly higher response rates to platinum-based regimens than non-carriers (22, 23). Collectively, these findings highlight the growing importance of prospective genomic profiling in guiding personalized chemotherapy.

A subset of PDAC cases arises from hereditary predisposition, primarily linked to germline mutations in genes such as BRCA1/2, ATM, PALB2, STK11, and DNA mismatch repair (MMR) genes (MLH1, MSH2, MSH6, PMS2) (24–28). Among these, BRCA2 mutations represent the most common inherited cause, accounting for 5–17% of hereditary cases (29, 30). Targeting such defects, poly(ADP-ribose) polymerase inhibitors (PARPis) have emerged as a key therapeutic strategy.

Mechanistically, PARP inhibitors trap PARP-1 on DNA and impair single-strand break (SSB) repair, leading to the accumulation of double-strand breaks (DSBs) and synthetic lethality in homologous recombination repair (HRR)-deficient tumors, such as those with BRCA1/2 mutations (31–33). Beyond DNA repair, PARP-1 participates in diverse oncogenic processes including chromatin remodeling, transcriptional regulation, hypoxia response, angiogenesis, and epithelial–mesenchymal transition (34).

Several PARPis, including olaparib, rucaparib, and niraparib, are approved clinically (35). In PDAC, olaparib is the most widely used PARPi, while rucaparib and talazoparib are also used as monotherapy (36). However, their utility is constrained by acquired resistance and treatment-related toxicities.

To address these issues, combination strategies are being actively explored. For instance, PARPis are being evaluated in combination with vascular endothelial growth factor receptors (VEGFR) inhibitors, c-MET inhibitors, MEK inhibitors, AKT inhibitors, and ATR inhibitors in ongoing clinical trials (e.g., NCT04764084, NCT02498613, NCT04005690, NCT03682289). Preclinical studies further demonstrate synergistic effects when PARPis are combined with histone deacetylases (HDAC) or bromodomain and extraterminal domain (BET) inhibitors, suggesting a promising avenue for overcoming resistance (37–40). Although targeted therapy could increase the efficiency of chemotherapy, it still faces challenges to the translation of basic research findings into clinical applications for PDAC. Key obstacles include the low prevalence of actionable mutations and poor drug penetration resulting from the dense stromal microenvironment.

### 2.3 Radiotherapy

Apart from the chemotherapy and targeted therapy, radiotherapy plays a crucial role in the multidisciplinary management of PDAC. The long−term results of the Selective Chemoradiation in Advanced LOcalised Pancreatic Cancer (SCALOP) trial have confirmed the superiority of chemoradiotherapy (CRT) in locally advanced pancreatic cancer (LAPC) (41). Recent advances in radiotherapy techniques continue to enhance treatment precision and efficacy. Compared with conventional 3D conformal radiotherapy, intensity−modulated radiotherapy (IMRT) has demonstrated a more favorable safety profile in PDAC treatment (42, 43). Stereotactic body radiotherapy (SBRT) has also shown considerable promise in overcoming the inherent radio−resistance of PDAC. A systematic review highlighted key advantages of SBRT, such as shortened treatment duration, improved overall survival, and enhanced 1−year locoregional control rates, supporting its role as a viable therapeutic option for inoperable disease (44). Furthermore, combining SBRT with FOLFIRINOX chemotherapy has been associated with increased rates of radical surgical resection in LAPC patients (45). Meanwhile, emerging techniques such as stereotactic magnetic resonance−guided adaptive radiotherapy (SMART) have shown encouraging preliminary outcomes for unresectable PDAC (46, 47).

However, radiotherapy in PDAC is limited by minimal overall survival benefit, inability to control systemic disease, dose constraints from adjacent radiosensitive organs, intrinsic radioresistance, and a narrow therapeutic window. These factors collectively restrict its therapeutic efficacy in PDAC.

### 2.4 Immunotherapy

Immunotherapeutic strategies for PDAC mainly include immune checkpoint blockade (ICB), adoptive cell therapy (ACT), and monoclonal antibody-based treatments (48). ICB has shown limited efficacy in PDAC, with anti-PD-1 and anti-cytotoxic T-cell lymphocyte-4 (anti-CTLA-4) trials reporting overall response rates (ORRs) of 0% and 3%, respectively, largely due to an immunosuppressive TME (49–51). The primary ACT being employed in treating PDAC encompasses chimeric antigen receptor (CAR) T-cell therapy, T-cell receptor (TCR)-engineered T-cell therapy, natural killer (NK)-cell therapy, and CAR NK-cell therapy. CAR T-cell therapy, known as a “living drug”, differs from traditional anti-tumor medications due to its capacity to multiply and endure within the body. Recent phase II clinical trial and preclinical studies have demonstrated encouraging, though limited, antitumor activity of CAR T-cell therapy in PDAC (52, 53). NK cells, integral to the innate immune system, are widely present in peripheral blood, bone marrow, and various organs. NK cells play a crucial role in monitoring and responding to cancerous or virus-infected cells within the immune system. CAR-NK therapy has also shown promising outcomes, with one clinical case reporting disease control for five months in a PDAC patient treated with CAR-NK cells (54). TCR-T cell therapy, which engineers T cells derived from peripheral blood mononuclear cells (PBMCs), has also demonstrated potential. A 67-year-old female with metastatic, treatment-refractory PDAC achieved objective tumor regression following TCR-T therapy (55). Chimeric antigen receptor macrophages (CAR-M) offer unique advantages for solid tumors, owing to their inherent tumor infiltration capacities, phagocytic function, and minimal risk of graft versus host disease (GVHD). Recently, Zheng et al. reported that intraperitoneal administration of CAR-M cells targeting c-MET (CAR-M–c-MET), derived from human monocytic THP-1 and hMDM cells, rapidly migrated to tumor sites and significantly inhibited tumor growth without notable side effects (56). Recently, several molecular targets have been identified for monoclonal antibody-based immunotherapy. Studies have reported that CLDN18.2, expressed in up to 60% of PDAC, represents a particularly promising therapeutic target (57). Furthermore, toripalimab, a humanized anti-PD-1 IgG4 monoclonal antibody, has exhibited encouraging antitumor activity in PDAC (58). However, the dense stromal structure and profound immunosuppression within the PDAC TME continue to hinder the overall effectiveness of immunotherapeutic strategies.

Immunotherapy offers distinct advantages over chemotherapy, targeted therapy and radiotherapy, including greater specificity, reduced unnecessary treatment, and a lower incidence of adverse effects. In the following four clinical trials, immunotherapy demonstrated favorable efficacy without reported adverse events. In a Phase I study (NCT05088889), PDAC patients were treated with ipilimumab and nivolumab. Another Phase I trial (NCT06411691) evaluated the combination of an mKRAS vaccine plus botensilimab and balstilimab in advanced PDAC. A Phase I/II study (NCT05927142) investigated durvalumab combined with rintatolimod in advanced PDAC. Furthermore, a Phase II trial (NCT01174121) included four treatment arms: CD8^+^-enriched tumor-infiltrating lymphocytes (TILs), unselected TILs, unselected TILs plus pembrolizumab administered prior to cell infusion, and unselected TILs plus pembrolizumab given at the time of disease progression, all applied in patients with advanced PDAC.

Despite recent advances, immunotherapy efficacy in PDAC remains severely limited by the immunosuppressive TME, which is shaped by KRAS mutations, impaired antigen presentation, dysfunctional cytotoxic T lymphocytes (CTLs), and the activity of cancer-associated fibroblasts (CAFs), myeloid cells, dendritic cells, B cells, and regulatory T cells (59). Consequently, strategies aimed at remodeling or overcoming the immunosuppressive TME have become a major focus for enhancing immunotherapy efficacy in PDAC.

**Figure 2:**
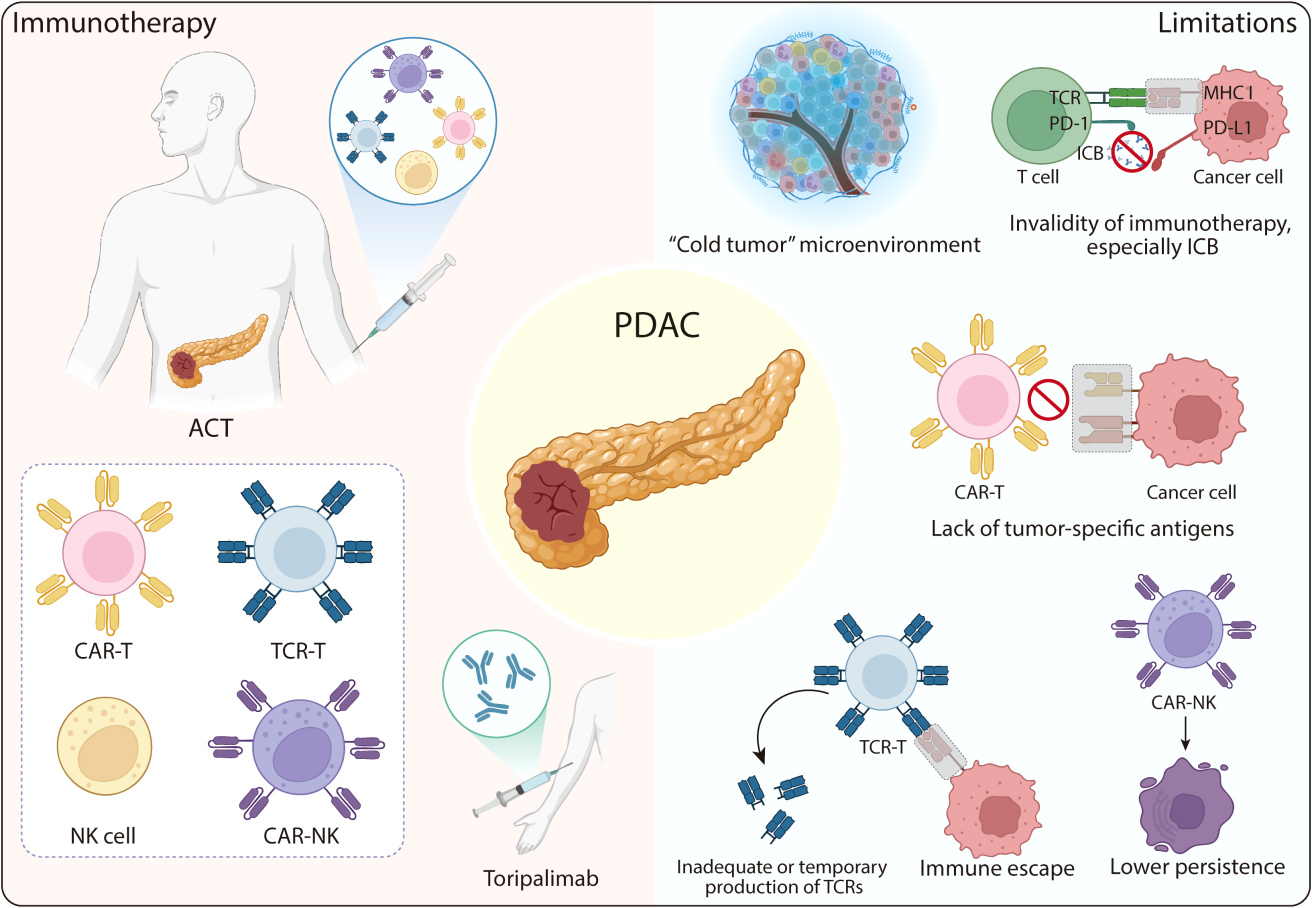
The current immunotherapy of PDAC and limitations. The immunotherapy of PDAC is primarily based on ACT, which includes CAR-T therapy, TCR-T therapy, NK therapy, and CAR-NK therapy. Additionally, toripalimab has shown promising therapeutic effects in PDAC treatment. Major obstacles in PDAC immunotherapy encompass a cold, immunosuppressive tumor microenvironment; the suboptimal performance of immunotherapies like ICB; the lack of tumor-specific antigens, which poses a significant barrier to CAR-T therapy. Regarding TCR-T therapy, its disadvantages include inadequate or temporary TCR production and immune escape. As for CAR-NK therapy, a key drawback is its lower persistence. ACT, adoptive cell therapy; ICB, immune checkpoint blockade; TCR: T cell receptor.

Each immunotherapeutic modality faces distinct challenges. CAR-T cell therapy is hindered by both the immunosuppressive TME and the scarcity of truly tumor-specific antigens. CAR-NK cells, while safer, often exhibit limited persistence *in vivo*, necessitating repeated infusion to sustain therapeutic efficacy. TCR-T cell therapy is constrained by suboptimal TCR expression and potential immune escape due to tumor antigen heterogeneity. Thus, overcoming the immunosuppressive TME represents a central goal for improving immunotherapy outcomes in PDAC (**Figure 2**).

Current therapeutic strategies for PDAC share common limitations, including single-target inhibition, dose-limiting toxicity, acquired drug resistance, and the immunosuppressive TME. These challenges underscore the need for multi-targeted agents with low toxicity, minimal drug resistance, and immunoregulatory properties.

## 3 The current and potential roles of miRNAs in PDAC therapy

MiRNAs are small, double-stranded non-coding RNAs (approximately 19–24 nucleotides in length) that regulate gene expression at the post-transcriptional level by promoting mRNA degradation or inhibiting translation of multiple target mRNAs. Unlike traditional therapies that typically inhibit a single protein, miRNAs offer the advantage of multi-target regulation (60). Nowadays, an increasing number of miRNA-based therapies have entered clinical trials for both diagnostic and therapeutic applications across a wide range of diseases. However, whether miRNA-based therapy has the potential for clinical trial in PDAC patients has not been discussed yet. Besides, miRNA-based therapy faces great challenges in clinical translation. In this part, we introduce the regulatory function of miRNAs in PDAC and clinical translation challenge that miRNA-based therapy faces.

### 3.1 The regulatory function of miRNAs in PDAC

In the context of miRNA-based therapy for PDAC, multiple miRNAs have demonstrated significant antitumor potential through targeted regulation of key oncogenic pathways. For instance, the restoration of p53-regulated miR-34b expression that often reduced in pancreatic tumors inhibits cell growth, induces cell cycle arrest, and enhances chemosensitivity via repression of the oncogene Smad3 (61, 62). Similarly, enforced expression of miR-34a targets anti-apoptotic Bcl-2, c-Myc, Cyclin D1, E2F3, and the Notch pathway, suppressing proliferation, metastasis, and invasion (63). Han et al. showed that overexpression of miR-455-3p impedes pancreatic cancer progression by inhibiting TAZ-mediated Wnt/β−catenin signaling and promotes apoptosis via modulation of Bcl-2 and Bax expression (64). miR−15a restoration similarly leads to cell cycle arrest and suppression of proliferation and epithelial−mesenchymal transition (EMT) through downregulation of Bmi−1 (65). Re−expression of miR−15b and miR−16 reduces Bcl−2 levels in pancreatic stellate cells and induces apoptosis (66). Furthermore, viral−mediated delivery of miR−143/145 or adenovirus−mediated expression of miR−143 inhibits tumor growth and metastasis in pancreatic cancer models (67, 68). Overexpression of miR-205 reduces stemness markers (OCT−3/4, NANOG) and pancreatic cancer stem cell markers (CD44, ALDH1) *in vitro* (69). Restoring miR−150 expression likewise suppresses malignant behavior and tumor growth, while re−expression of miR−137 attenuates migration and invasion by targeting the upregulated oncogenic factor MRGBP (70, 71). A defining feature of miRNAs is their ability to simultaneously regulate multiple oncogenic pathways, which distinguishes them from conventional single-target therapeutic strategies.

### 3.2 The adaption of miRNA-based therapy in PDAC and privilege of miRNA-based therapy

Aberrant miRNA expression is a hallmark of PDAC, with numerous miRNAs found to be downregulated and oncogenic miRNAs upregulated in tumor tissues. Therapeutically, miRNA-based strategies can be broadly categorized into two approaches: miRNA replacement and miRNA inhibition. The first involves miRNA mimics, which restore the expression of tumor-suppressive miRNAs that are lost during carcinogenesis. These mimics are double-stranded RNA molecules chemically modified *in vitro* to emulate endogenous miRNAs and reestablish their regulatory function (60). Preclinical studies have demonstrated promising efficacy, for example, viral-mediated delivery of miR-143/145 significantly suppresses tumor growth in PC models (68). The first miRNA therapeutic to enter clinical trials, MRX34 (a synthetic miR-34a mimic), demonstrated promising antitumor activity but was terminated due to severe immune-related toxicity, yet it provided critical proof-of-concept for clinical translation of miRNA-based therapeutics (72). Another approach targets oncogenic miRNAs that are aberrantly upregulated in PDAC and promote tumor growth, invasion, and therapy resistance (73). Several inhibitory strategies have been developed to silence aberrantly upregulated miRNAs, including antisense anti-miR oligonucleotides (AMO), locked nucleic acid (LNA), miRNA antagomirs, and miRNA sponges. For example, Griveau et al. *in vivo* study showed that LNA-modified antisense oligonucleotides effectively silenced overexpressed miR-21 in glioblastoma models, leading to reduced cell viability and increased caspase activation (74). Beyond direct oncogenic regulation, many miRNAs also play critical roles in drug and radio-resistance in PDAC (75, 76). For example, Gu et al. identified hsa-miR-3178 as a promoter of gemcitabine resistance, suggesting its inhibition could restore chemosensitivity (77). Similarly, Wang et al. reported that miR-23b overexpression sensitized PC cells to radiation (78). Collectively, these findings underscore the potential of miRNAs as both standalone therapeutic agents and adjuvants to conventional chemotherapy or radiotherapy in PDAC.Compared to conventional treatments, miRNA-based therapies offer several distinct advantages. First, by modulating multiple functionally interconnected genes, miRNAs target functional networks rather than isolated pathways. This broader mechanism of action enables more comprehensive suppression of tumor-promoting processes and reduces the likelihood of resistance development.

Second, unlike traditional therapies, which often induce resistance through mechanisms such as enhanced DNA repair, evasion of apoptosis, or target gene mutations, and are associated with toxicities including myelosuppression, gastrointestinal injury, and organ damage, miRNA-based strategies offer a more controlled approach. However, resistance to miRNA-based strategies may involve alterations in drug-target genes, pharmacokinetic genes, related signaling pathways, or DNA damage repair mechanisms. Toxicity in miRNA-based therapies primarily stems from off-target effects and on-target over-suppression, the latter referring to adverse outcomes due to excessive inhibition of the intended target genes.

Finally, another major advantage of miRNA-based therapeutics lies in precision medicine. Whereas conventional therapies rely on clinicopathological features, molecular biomarkers, pharmacokinetics, and prognostic indicators, miRNA-based treatment can be guided directly by miRNA expression levels in tumor tissues. For instance, in pancreatic cancer, patients with low expression of specific tumor-suppressive miRNAs may benefit from miRNA mimics, while those with overexpression of oncogenic miRNAs could be candidates for miRNA inhibitors, enabling a more personalized and mechanism-driven therapeutic paradigm.

Collectively, although miRNA-based therapies are still in early clinical development, accumulating preclinical and clinical evidence support their potential as innovative and versatile therapeutic modalities for pancreatic cancer, offering mechanistic advantages that complement, and in certain contexts may surpass, those of conventional treatment strategies.

### 3.3 Integrating miRNA-based and conventional therapies in PDAC

Although miRNA-based therapy has demonstrated superior efficacy to traditional therapy in preclinical studies, and combination approaches have been shown to enhance therapeutic efficacy while reducing chemoresistance, the clinical translation of miRNA therapeutics remains challenging. Notably, miRNA-based therapy complements conventional treatment modalities in three key areas: chemotherapy, radiotherapy, and PARP inhibitor therapy.

Gu et al. found that miR-3178 upregulates the expression of ATP-binding cassette (ABC) transporter proteins via the RhoB/PI3K/Akt pathway, thereby promoting gemcitabine (GEM) resistance (77). This finding suggests miR-3178 as a novel target for enhancing chemotherapy sensitivity in PDAC. Liu et al. revealed that miR-3662 regulates PC cell metabolism through a HIF-1α negative feedback loop, reversing GEM resistance, indicating that combining miR-3662 with GEM could be a promising therapeutic strategy (79). Ouyang et al. reported that miR-499a-5p is overexpressed in 5-FU-resistant PC cells, promoting resistance and proliferation by targeting PTEN and activating the PI3K/Akt pathway; inhibiting miR-499a-5p enhanced apoptosis and chemotherapy efficacy (80). Lee et al. showed that blocking miR-1976 increases chemosensitivity by promoting the apoptosis gene XAF1, suggesting that a miR-1976 inhibitor combined with chemotherapy could enhance anti-tumor effects (81). Similarly, upregulating miR-31-5p has been associated with chemoresistance in PC cells (82).

Zhang et al. established a radiotherapy-resistant PC cell line and observed significant downregulation of miR-216a. Further studies demonstrated that miR-216a overexpression directly targets the 3′-UTR of beclin-1, inhibiting PC cell growth and promoting apoptosis in resistant cells, indicating its potential to enhance radiotherapy efficacy through autophagy and apoptosis modulation (83). McGrath et al. showed that regulating miR-31 alters GPx8 expression, increasing PC cell radiosensitivity (84). Other miRNAs such as miR-216b, miR-23b, miR-374, miR-296-3p, miR-6855-5p, and miR-26a (upregulated) or miR-620 and miR-99b (inhibited) have also been identified as potential radiosensitizers in PC (76, 78, 85–90).

MiRNAs influence cellular response to PARPis by modulating genes involved in HRR and base excision repair (BER) (91). For example, miR-182, miR-155, and miR-30a regulate BRCA1/2, RAD51, and other DNA repair factors, affecting PARPi sensitivity. Some miRNAs, like miR-34a, directly target PARP1 (92). Additionally, miRNAs can mediate PARPi resistance through other mechanisms: miR-181a promotes resistance in triple-negative breast and ovarian cancers by downregulating the STING pathway, while miR-622 confers resistance via Ku70/Ku80 interaction, impairing non-homologous end joining (NHEJ) (93, 94).

These findings strengthen the rationale for developing miRNAs as therapeutic candidates, highlighting their potential to enhance traditional cancer treatments through multi-target network regulation and personalized stratification.

### 3.4 The function of miRNAs targeting TME in PDAC

Previous studies have demonstrated the advantages of miRNA-based therapy and combination therapy in enhancing efficacy and reducing drug resistance. MiRNAs could also affect various molecular and cellular components within the TME, representing an additional therapeutic avenue for immunotherapy in PDAC. These immune-modulatory miRNAs (im-miRNAs) are critically involved in mediating immune escape.

Mutations in KRAS, present in approximately 92% of PDAC cases, drive the recruitment of immunosuppressive myeloid cells, induce immunosuppressive Th17 and gamma-delta T cells, and upregulate programmed death ligand-1 (PD-L1) expression (95–97). miRNAs like let-7, miR-18a, miR-31, and miR-143 regulate KRAS gene expression and serve as potential therapeutic targets against KRAS-driven immunosuppression in PDAC (98).

CAFs are key contributors to the desmoplastic and fibrotic TME. They secrete factors that promote tumorigenesis and suppress antitumor immunity (99). Meanwhile, CAFs appear to limit the migration of CTLs to the stromal compartments through activation of focal adhesion kinase (FAK) as well as diminishing CTLs function via soluble immunosuppressive mediators such as interleukin-10 (IL-10), transforming growth factor-β (TGF-β), vascular endothelial growth factor (VEGF), prostaglandin E1, IDO, arginase, and expression of PD-L1 (100). Numerous miRNAs have been documented to influence CAF gene expression across various tumors (101). Zhou et al. engineered integrin α5-targeting peptide-modified extracellular vesicles (IEVs-PFD/138) loaded with miR-138-5p and the anti-fibrotic agent pirfenidone (PFD) to reprogram CAFs and inhibit their pro-tumorigenic effects. This is achieved through miR-138-5p-mediated downregulation of FERMT2 in CAFs, which suppresses TGF-β receptor (TGFBR1) signaling and subsequently reprograms CAFs (102). Similarly, Qi et al. reported that miR-3173-5p, derived from CAF exosomes, targeted ACSL4 in PDAC cells after uptake, leading to decreased ACSL4 expression and consequently reduced intracellular Fe²^+^ and lipid reactive oxygen species (ROS) levels, which in turn inhibited ferroptosis (103). Although more studies about the function of miRNAs on CAFs in PC have not been reported, miRNAs present a promising therapeutic strategy for targeting CAFs and reprogramming the TME in combination with immunotherapy.

Antigen presentation is another crucial component of immune evasion in PDAC. Both classical MHC-I surface antigens induced APM components and non-classical MHC-I molecules (HLA-G and -E) are often altered, facilitating escape from T cell- and NK cell-mediated cytotoxicity. Several miRNAs have been implicated in modulating MHC-I molecule expression. For example, miR-125a targets MHC-I in esophageal adenocarcinoma cell lines, leading to reduced levels of MHC-I required for antigen presentation, which in turn suppresses the anti-tumor immune response and results in poor patient prognosis (104). In addition, miR-138-1-3p targets HLA-G90 to inhibit the progression of papillary thyroid carcinoma (PTC) (105). Although miRNAs exert vital functions on antigen function in TME, and such impairment is always associated with immunotherapy resistance, their precise roles in modulating antigen presentation in PC remain largely unexplored and represent a compelling direction for future research.

**Figure 3:**
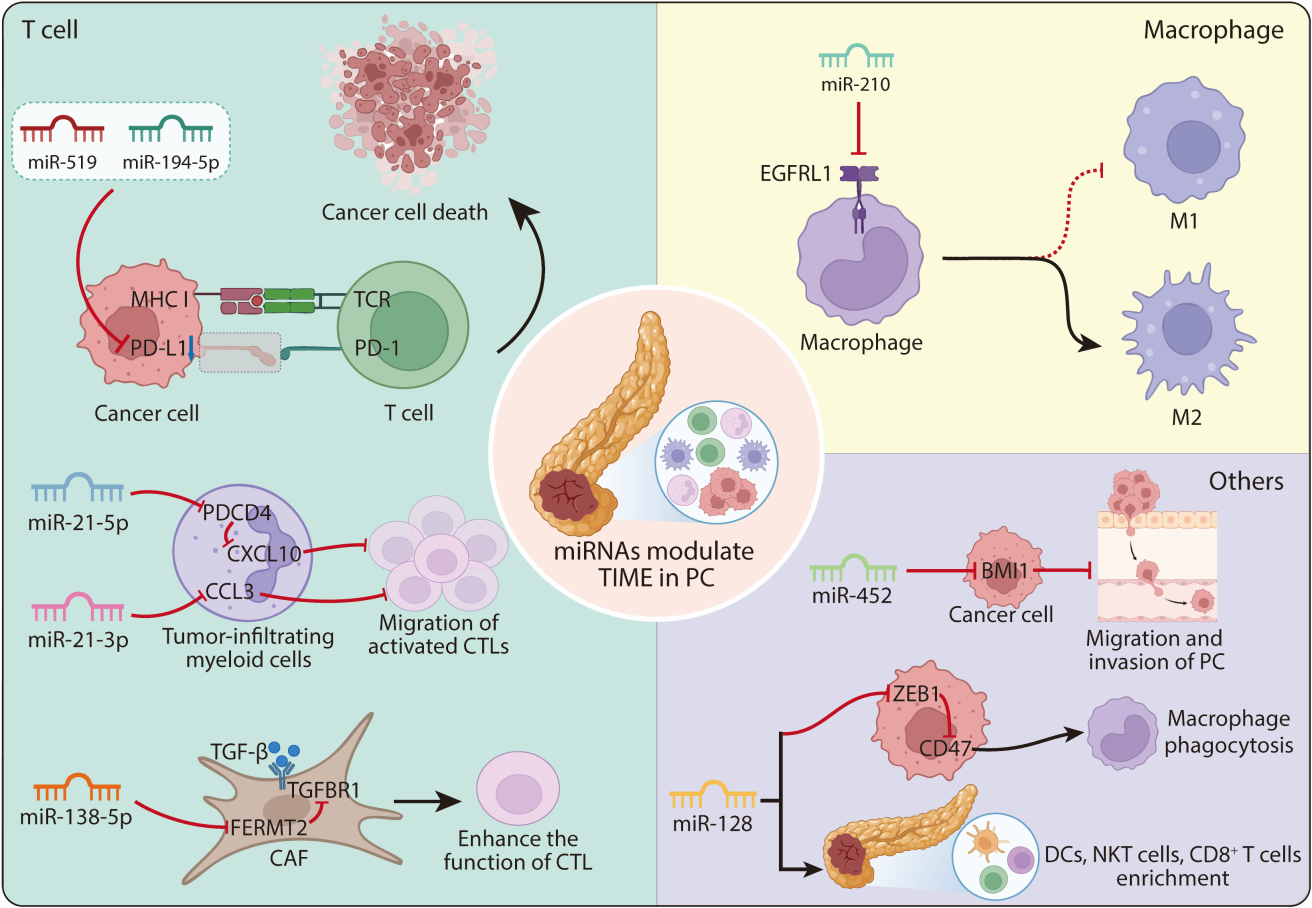
Exogenous miRNAs modulate tumor immune microenvironment in PC. Based on their mechanisms of action, exogenous miRNAs can modulate the TIME in PC through four primary pathways: (1) T cell-related regulation. MiR-519 suppresses immune escape by downregulating PD-L1 expression. MiR-194-5p inhibits CD8^+^ T cell efficacy via PD-L1 suppression. In tumor-infiltrating myeloid cells, miR-21-5p inhibits CXCL10 by targeting PDCD4, whereas miR-21-3p directly inhibits CCL3; together, these two pathways impair the migration of activated CTLs within the PC TME. MiR-138-5p inhibits the FERMT2-TGFBR1-TGF-β signaling pathway, thereby suppressing CAF-mediated impairment of CTL function. (2) Macrophage-related regulation. MiR-210 facilitates the polarization of tumor-associated macrophages toward the M2 phenotype by inhibiting EGFRL1 expression. (3) Other mechanisms: MiR-452 impedes PC progression by targeting BMI1. MiR-128 inhibits CD47 by inhibiting ZEB1 in tumor cells. This leads to enhanced macrophage phagocytosis. Besides, miR-128 increases the proportions of DCs, CD8^+^ T cells, and NKT cells within both the tumor microenvironment and the spleen in PC models. TIME, tumor immune microenvironment; PC, pancreatic cancer; CAFs, cancer-associated fibroblasts; DCs, dendritic cells; NKT cells, natural killer T cells; CTLs, cytotoxic T lymphocytes.

The PDAC TME is largely composed of CD45^+^ bone marrow–derived immune cells, particularly myeloid-derived suppressor cells (MDSCs) and tumor-associated macrophages (TAMs) (106, 107). Therapeutic strategies targeting these populations, such as CXCR2 inhibition or TAM repolarization, have shown enhanced ICB efficacy in preclinical models (108). Preclinical studies have identified that dual inhibition of both TAMs and MDSCs can repolarize M2 TAMs towards an antitumor M1 phenotype, leading to improved survival (109). Accumulating evidence highlights the pivotal role of miRNAs in regulating the immunosuppressive functions of these myeloid populations. For example, miR-21 is significantly upregulated in myeloid cells surrounding both pancreatic intraepithelial precursor lesions (PanIN) and invasive cancers. In this context, miR-21-5p suppresses CXCL10 via PDCD4 inhibition, while miR-21-3p directly inhibits CCL3; both axes converge to impair the migration of activated CTLs within the PC TME. This suppressive activity is localized to tumor-infiltrating myeloid cells, where these molecular interactions collectively orchestrate the remodeling of the immune landscape (110). Binenbaum et al. demonstrated that introducing miR-365 into TAMs led to gemcitabine resistance in PDAC-bearing mice, while transferring a miR-365 antagonist restored gemcitabine sensitivity (111). Moreover, miRNAs could also regulate the polarization between M1 and M2 TAMs (112). The report indicates miR-210 could induce the M2 polarization of TAMs in the PC TME by suppressing FGFRL1 expression in macrophages (113). However, more detailed mechanistic studies on miRNA regulation within myeloid cells need to be discussed further in PC. Moreover, studies have demonstrated that overexpression of miR-128 in tumor cells enhance the infiltration of dendritic cells (DCs), CD8^+^ T lymphocytes, and natural killer T (NKT) cells in the tumors and spleens, consequently enhancing antitumor immunity in PDAC mouse models. Beyond its role in promoting immune cell infiltration, miR-128 also inhibit CD47 by inhibiting ZEB1 in PDAC cells, further enhancing macrophage phagocytosis in the PDAC TME (114). In addition, some reports indicated miRNAs could also regulate the CD8^+^ T cell function (115). For instance, miRNA let-7 enhances memory CD8^+^ T-cell formation and aids in the clearance of melanoma. Additionally, let-7 promotes the activity of CTLs and memory CD8^+^ T cells in the TME of melanoma to exert antitumor effects by inhibiting the mTOR/ROS axis (116). Other miRNAs could exert other functions. For instance, miR-452 was reported to directly target B-cell-specific Moloney murine leukemia virus insertion site 1 (BMI1) in PC cells, and its overexpression suppressed tumor migration and invasion (117). These results indicated miRNAs could regulate DC infiltration and B cell function, providing additional opportunities for combinatorial immunotherapy approaches. Finally, miRNAs also influence chemokine signaling and metabolic pathways in TME (118, 119). Collectively, these studies highlight miRNAs as multifaceted regulators of immune and stromal components in PDAC, offering promising targets to improve immunotherapy efficacy (**Figure 3**).

### 3.5 The role of miRNAs in regulating immunotherapy

The immunomodulatory functions of miRNAs in cancer treatment mainly center on regulating ICB molecule expression and enhancing the efficacy of CAR-T and CAR-NK therapies. Although ICB has shown limited effectiveness in PDAC, largely due to the immunosuppressive TME, the expression levels of ICB-related molecule remain a critical determinant of therapeutic efficacy. Numerous miRNAs have been reported to modulate ICB molecules through post-transcriptional regulation (120). For instance, members of the miR-15 family, including miR-15a and miR-15b, target PD-L1 in neuroblastoma and lung adenocarcinoma, thereby enhancing NK and CD8^+^ T-cell activity (121). Likewise, CD86 ligand of the cytotoxic T lymphocyte-associated protein-4 (CTLA-4) could be regulated by miR-20b-5p in renal cell carcinoma (RCC) (122). In PC, several miRNAs, such as miR-194-5pand miR-519 have been reported to target PD-L1, influencing tumor progression and immune escape (123, 124). Additionally, miR-148a-3p exerts a similar role in colorectal cancer (125). However, the detailed molecular mechanisms of these interactions remain incompletely elucidated, and the role of miRNAs in regulating other immune checkpoint molecules in PDAC is still largely unexplored.

**Figure 4:**
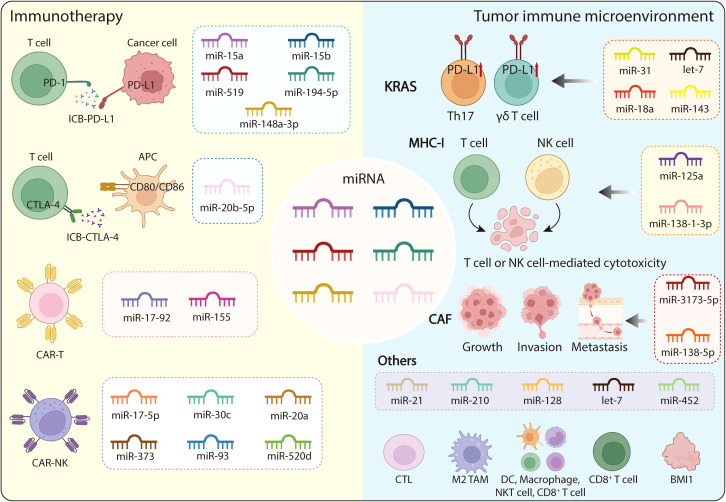
The regulatory function of miRNA immunotherapy and tumor microenvironment. The field of miRNA-based immunotherapy primarily encompasses the following three strategies: ICB, CAR-T therapy, and CAR-NK therapy. This includes blocking PD-L1 and CTLA-4 for ICB. Several miRNAs, such as miR-15a, miR-15b, miR-194-5p, miR-148a-3p, and miR-519, can target PD-L1, while miR-20b-5p can regulate CTLA-4. In CAR-T therapy, the function and infiltration of CAR-T cells are influenced by miRNAs like miR-17–92 and miR-155. For CAR-NK therapy, miRNAs such as miR-30c, miR-17-5p, miR-20a, miR-93, miR-373, and miR-520d affect its activities. The tumor microenvironment can be altered through various means, including mutating the KRAS and CAF genes, targeting MHC-I, among others. Numerous miRNAs can regulate key players like KRAS, CAF, MHC-I, T cells, TAMs, and MDSCs. For instance, Let-7, miR-18a, miR-31, and miR-143 target KRAS, thereby influencing the induction of immunosuppressive Th17 and γδ T cells, and the upregulation of PD-L1 expression. miR-138-5p and miR-3173-5p target CAF, which promotes tumor growth, invasion, and metastasis. miR-125a and miR-138-1-3p target MHC-I, thus affecting T cell-mediated and/or NK cell-mediated cytotoxicity. miR-21 can impair migration of activated CTLs. miR-210 can induce the polarization of M2 TAMs. miR-128 can increase the percentages of DCs, CD8^+^ T cells, and NKT cells and macrophage phagocytosis. Let-7 can promote the formation of memory CD8^+^ T cells and activity of CD8^+^ T cells. miR-452 can target BMI1. ICB, immune checkpoint blockade; MDSC, myeloid-derived suppressor cell; TAM, tumor-associated macrophage; CTLs, cytotoxic T lymphocytes.

Beyond ICB regulation, miRNAs also play pivotal roles in modulating the function, persistence and safety of CAR-T cells. They can mitigate cytokine release syndrome (CRS) and neurotoxicity by reducing the release of cytokines (e.g., IL-6 and IFN-γ), promote the formation of a memory-like phenotype associated with sustained antitumor activity and reduced toxicity, enhance CAR-T cell infiltration into tumors, and drive the production of proinflammatory cytokines (126). In CAR-T therapy, persistence of effector T cells is promoted by miR-17–92 and miR-155 (127, 128). Similarly, overexpression of miR-155 has also been shown to enhance T-cell activity against solid tumors (129). Collectively, these findings suggest that miRNAs could positively regulate CAR-T cell performance; therefore, combining miRNA modulation with CAR-T therapy may further enhance its therapeutic effectiveness. In the context of CAR-NK therapy, miRNAs are also involved in regulating NK recognition receptors, thereby influencing the activities of CAR-NK therapy. For instance, transfection with miR-30c upregulates the NK activation receptor NKG2D by targeting the inhibitory transcription factor HMBOX1, thus enhancing antitumor responses (130). NKG2D recognizes eight ligands (NKG2DLs), including major histocompatibility complex class I chain A (MICA) and MICB. Conversely, overexpression of miR-17-5p, miR-20a, miR-93, miR-373, and miR-520d has been shown to downregulate MICA, resulting in decreased NK cell-mediated cytotoxicity (131). In glioma cells, inhibiting miR-93, an NKG2D ligand-targeting miRNA, enhanced NK cell-mediated cytotoxicity, underscoring miRNAs’ role in innate immune evasion (132). However, whether similar miRNA-mediated regulatory mechanisms influence CAR-NK activity in PDAC remains to be elucidated. In summary, miRNAs can profoundly affect immune checkpoint expression and the functional dynamics of CAR-based cell therapies. Targeting or harnessing miRNAs therefore represents a promising approach to enhance the efficacy of various immunotherapeutic modalities in PDAC. Future research should investigate the molecular mechanisms underlying these regulatory effects and explore their potential as combinatory agents in next-generation immunotherapeutic strategies (**Figure 4**) (**Table 1**).

**Table 1:** The microRNAs on tumor immunotherapy.

Species	Type	Roles and outcomes	Mechanisms	References
miR-15a, miR-15b	TsmiR	Promote natural killer and CD8^+^ T cell activation and anti-tumor immune response in neuroblastoma	Inhibit PD-L1	(121)
miR-20b-5p	TsmiR	Increase immune infiltration in the tumor microenvironment of kidney renal clear cell carcinoma	Increase expression of CTLA-4	(122)
miR-194-5p	TsmiR	Inhibit the progression of pancreatic cancer and boost the anti-tumor effect of CD8^+^ T cells	Inhibit PD-L1	(124)
miR-148a-3p	TsmiR	Inhibit the progression of colorectal cancer and boost the anti-tumor effect of CD8^+^ T cells	Inhibit PD-L1	(125)
miR-519	TsmiR	Inhibit the progression of pancreatic cancer	Inhibit PD-L1	(123)
miR-17-92	TsmiR	Inhibit the progression of glioblastoma	Promote persistence of effector T cells	(128)
miR-155	TsmiR	Improve anti-tumor activity in B16 tumors	Promote both proliferation and effector functions of CD8+ T cells	(127)
miR-155	TsmiR	Promote the expression of AKT and Stat5a,enhance T-cell responsiveness to limited amounts of homeostatic γc cytokines in melanoma	Enhance T-cell activity against solid tumors	(129)
miR-30c	TsmiR	Promote the cytotoxicity of NKL cells	Upregulate the expression levels of NKG2D, CD107a and FasL	(130)
let-7, miR-18a, miR-31, miR-143	TsmiR	Inhibit cancer	Inhibit KRAS	(98)
miR-138-5p	TsmiR	Inhibit pancreatic cancer	Inhibit FERMT2-TGFBR1-TGF-β signaling pathway to inhibit CAF	(102)
miR-3173-5p	TsmiR	Inhibit chemoresistance induced by Gemcitabine in pancreatic cancer	Sponge ACSL4 and inhibit ferroptosis after uptake by cancer cells	(103)
miR-138-1-3p	TsmiR	Inhibit the progression of papillary thyroid carcinoma	Target HLA-G90	(105)
miR-128	TsmiR	Enhance macrophage phagocytosis in the TME of pancreatic cancer	Inhibit CD47 by increasing the expression of ZEB1	(114)
let-7	TsmiR	Promote the formation of memory CD8^+^ T cells, activity of CTLs and memory CD8^+^ T cells in B16^gp33^ tumor-bearing mice	Inhibit the PI3K/AKT/mTOR signaling pathway and production of reactive oxygen species	(116)
miR-452	TsmiR	Inhibit the migration and invasion of pancreatic cancer	Inhibit BMI1	(117)
miR-21	OncomiR	Promote the progression of pancreatic cancer	Induce the generation of MDSCs	(110)
miRNA-21-5p	OncomiR	Impair migration of activated CTLs in the TME of pancreatic cancer	Inhibit expression of CXCL10 in tumor-infiltrating myeloid cells	(110)
miRNA-21-3p	OncomiR	Impair migration of activated CTLs in the TME of pancreatic cancer	Inhibit expression of CCL3 in tumor-infiltrating myeloid cells	(110)
miR-125a	OncomiR	Suppress the anti-tumor immune response in esophageal adenocarcinoma	Reduce levels of MHC-I required for antigen presentation	(104)
miR-210	OncomiR	Induce the M2 polarization of TAMs in pancreatic cancer	Inhibit the levels of FGFRL1	(113)
miR-17–5p, miR-20a, miR-93, miR-373, miR-106b, miR-520d	OncomiR	Downregulate MICA accompanied by a decreased NK cell susceptibility	Avoid NKG2D-mediated MICA immune recognition	(137)

### 3.6 Clinical translation potential and obstacle of miRNA-based therapies in PDAC

MiRNA-based therapy exhibits great advantages in chemotherapy and exerts a complementary role in immunotherapy. Therefore, advancing miRNA-based therapy into clinical translation is of great significance. Based on the functions of oncogenic miRNAs (oncomiRs) and tumor-suppressive miRNAs (tsmiRs), two primary modalities have been developed in miRNA-based drug therapy: miRNA mimics (chemically modified double-stranded RNAs) and miRNA inhibitors (single-stranded RNAs). miRNA mimics are designed to supplement tsmiRs, thereby restoring their natural regulatory function. Conversely, miRNA inhibitors are engineered to bind complementarily to oncomiRs, effectively blocking their activity.

The first miRNA-based cancer therapy to enter clinical trials was MRX34, a synthetic mimic of the tumor suppressor miR-34a, which commenced its phase 1 study (NCT01829971) in 2013. miR-34a functions as a tumor suppressor primarily through selective targeting of the p53 protein, and its expression is frequently downregulated across various cancers. Preclinical studies demonstrated that miR-34a effectively inhibits proliferation, induces apoptosis, and suppresses metastasis and invasion, with no observable toxicity in animal models, prompting efforts to evaluate its clinical translatability (61). In the phase I trial conducted by Beg et al., MRX34 was administered intravenously twice weekly to patients with advanced solid tumors, including pancreatic cancer, demonstrating initial evidence of anticancer activity. However, despite its promising therapeutic potential, the trial was halted three years later due to severe immune-mediated toxicities and patient deaths, underscoring the challenges of clinical translation for miRNA-based agents (72, 133). Several key factors likely contributed to this outcome. From a delivery standpoint, the liposomal formulation of MRX34 effectively protected the miRNA from degradation, extended its circulation half-life from minutes to hours, promoted tumor accumulation via the enhanced permeability and retention (EPR) effect, and facilitated cellular uptake through endocytosis or membrane fusion. However, this system lacked active tumor-targeting capability, resulting in widespread biodistribution. Intravenous administration of MRX34 in mice and non-human primates resulted in substantial accumulation in the liver, bone marrow, spleen, and lung, indicating broad off-target biodistribution in preclinical studies (72). This lack of delivery specificity likely contributed to the premature termination of the trial. Additionally, the trial design lacked adequate patient stratification: no selection was performed based on p53 status, miR-34a expression levels, or its target gene signatures, nor was stratification by tumor subtype implemented. Thus, while MRX34 validated the feasibility and antitumor potential of miRNA mimics, its clinical development was ultimately limited by systemic toxicity, highlighting the need for improved delivery systems and toxicity management in future miRNA-based therapeutics.

In contrast, TTX-MC138, an inhibitor of oncogenic miR-10b, represents an advanced delivery strategy. It consists of a miR-10b inhibitor conjugated to ultrasmall iron oxide nanoparticles coated with dextran. MiR-10b promotes tumor metastasis and is upregulated in PDAC compared with normal tissues (134, 135). Preclinical studies demonstrating its efficacy and favorable safety profile in animal models provided the foundation for clinical translation. In a preclinical study by Ghosh et al., weekly intravenous administration in mice bearing orthotopic PC xenografts significantly reduced tumor growth, with 40% of tumors showing complete regression and no recurrence or metastasis observed 10 weeks post-treatment (136). Preliminary phase I data (NCT05908773) suggest favorable tolerability, prolonged circulation, and accumulation in metastatic lesions. TTX-MC138 addresses several limitations of earlier miRNA therapies: its dual-targeting strategy enables active targeting of tumor tissues and cells, surpassing the passive EPR-dependent accumulation of MRX34. This approach enhances delivery precision, reduces off-target effects, minimizes immune activation through optimized nanoparticle design, and improves pharmacokinetic profiles. In addition to its advanced delivery system, the trial design incorporated rigorous patient stratification, a key factor distinguishing it from MRX34. The phase I study enrolled only patients with metastatic tumors, effectively enriching the population for individuals with high miR-10b expression, given the established role of miR-10b as a driver of metastasis.

Despite such advances, the development of miRNA-based therapies faces several critical challenges, including toxicity, off-target effects, and unintended immune activation, which are further complicated by disparities between preclinical models and clinical outcomes as well as pharmacokinetic barriers. Toxicity primarily arises from on-target over-suppression, where excessive inhibition of intended genes disrupts essential biological pathways, and from off-target effects due to the promiscuous binding capacity of miRNAs. Because miRNAs recognize target mRNAs through short seed sequences, a single miRNA can interact with hundreds of transcripts, often with partial complementarity, leading to unintended gene silencing—particularly problematic in the complex and dysregulated gene networks of human cancers. Immune activation is another significant concern, as exogenous RNA molecules can be detected by innate immune receptors such as Toll-like receptors and RIG-I, triggering inflammatory cytokine release and potentially severe immune-mediated adverse events, as observed in the MRX34 trial.

These issues are exacerbated by the translational gap between animal studies and human applications. Discrepancies arise from fundamental differences in miRNA expression profiles and regulatory networks between model organisms and humans, the greater complexity of the human tumor microenvironment and immune interactions, the polygenic and heterogeneous nature of human diseases, and species-specific variations in pharmacokinetics and immune responses. Such differences underscore the limitations of current preclinical models and highlight the need for more physiologically relevant systems. Pharmacokinetic hurdles further impede progress: unprotected RNAs are rapidly degraded by ribonucleases, resulting in a short circulatory half-life, while unmodified miRNAs struggle to cross cell membranes, are quickly cleared by the kidneys, or accumulate in reticuloendothelial organs like the liver and spleen, limiting their bioavailability in target tissues. The absence of efficient and safe delivery systems compounds these challenges, often leading to low targeting efficiency, carrier-related toxicity, and difficulties in scaling up production. Together, these factors illustrate the multifaceted obstacles that must be addressed to advance miRNA therapeutics from bench to bedside.

In summary, miRNA-based therapies hold significant potential for pancreatic cancer treatment, as evidenced by iterative improvements from MRX34 to TTX-MC138. Realizing this potential will require continued innovation in targeted delivery, immune-compatible design, and physiologically relevant preclinical models to overcome the intertwined challenges of toxicity, specificity, and translational fidelity.

## 4 Delivery strategies of miRNAs for PDAC treatment

**Figure 5:**
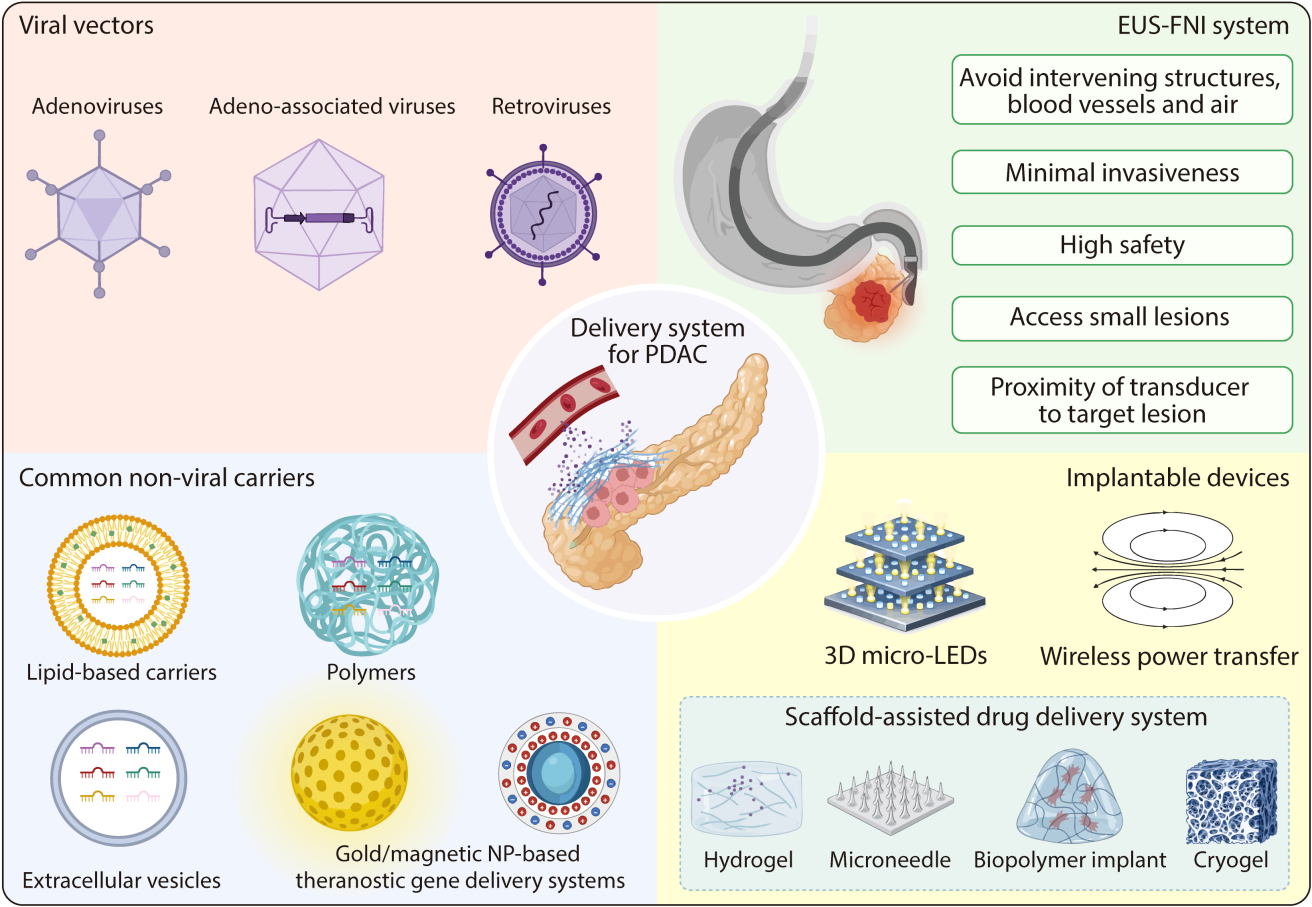
The delivery system adapted on PDAC. Delivery systems adapted for PDAC include viral vectors, common non-viral carriers, the EUS-FNI system, and implantable devices. Viral vectors comprise adenoviruses, adeno-associated viruses, and retroviruses. Common non-viral carriers include lipid-based carriers, extracellular vesicles, polymers, and gold/magnetic nanoparticle-based theranostic gene delivery systems. The EUS-FNI system offers several advantages: avoiding intervening structures, blood vessels, and air; minimal invasiveness; high safety; access to small lesions; and proximity of transducer to the target lesion. Implantable devices include 3D micro-LEDs, wireless power transfer, and scaffold-assisted drug delivery system. Among these, scaffold-assisted drug delivery systems encompass hydrogel, microneedle, biopolymer implant, and cryogel. They function by delivering immunotherapeutic agents. EUS-FNI, endoscopic ultrasound-guided fine needle injection.

The clinical failure of miRNAs indicates that improving the precision of miRNA delivery is essential to reduce side effects. To address this, a variety of delivery strategies have been developed. Apart from viral and non-viral vector delivery systems, more precise and flexible platforms, such as EUS-guided fine needle injection (EUS-FNI) and implantable devices, have also been established, thereby advancing the clinical translation of miRNA-based therapy (**Figure 5**).

### 4.1 Therapeutic strategies for miRNA delivery

Accumulating evidence highlights the therapeutic potential of miRNAs in PDAC, emphasizing the urgent need for efficient delivery systems capable of penetrating the dense, fibrotic tumor stroma to achieve sufficient intratumoral concentrations and exert antitumor activity. Both viral and non-viral vectors have been explored for miRNA delivery. Viral vectors after detoxification are commonly employed to deliver non-coding RNAs to cancer cells, including adenoviruses (ADs), adeno-associated viruses (AAVs), and retroviruses (RVs) (138). However, their inherent toxicity, immunogenicity, and limited loading capacity restrict their clinical translation. To overcome these challenges, various non-viral carriers have been developed, including lipid-based nanoparticles, polymeric systems, and gold/magnetic nanoparticle(NP)-based theranostic gene delivery systems. Li et al. developed a targeted co-delivery NP system carrying miRNA-21 antisense oligonucleotides (ASO-miR-21) and gemcitabine (GEM), which demonstrated synergistic inhibition of PC cell metastasis and tumor growth (139). Zhang et al. reported that ionizable cationic lipid nanoparticles (LNPs) are an effective carrier for miRNA-125b and significantly inhibited M2 macrophage polarization in PC treatment (140). Xin et al. synthesized a block copolymer (mPEG-co-P(Asp)-g-TEPA-g-DC) capable of forming stable complexes with miRNAs and further enhanced miR-519c stability and activity through 2’-O-methyl phosphorothioate (OMe-PS) modification at the 3’ end, which prevented immunogenicity (141). The multifunctional nanomedicine of GEM and OMe-PS-miR-519c offers a novel therapeutic strategy to treat desmoplasia and hypoxia-induced chemoresistance in PC. In addition, exosome-based delivery systems have attracted growing interest due to their intrinsic biocompatibility, immune evasion capability and can evade macrophage phagocytosis and lysosomal degradation. Zuo et al. isolated exosomes from cells and used ultrasonic methods to synthesize miR-34a coated exosomes (exomiR-34a), which effectively penetrated cell membranes and significantly inhibited PC development both *in vitro* and *in vivo* (142). While Song Shang investigated EV-derived miRNA-1231 and observed that its low expression correlates significantly with advanced TNM staging in pancreatic cancer and promotes cancer cell proliferation and migration. Experiments showed that exogenous administration of bone marrow mesenchymal stem cell (BM-MSC)-derived miRNA-1231 to pancreatic cancer cell lines (BxPC-3 and PANC-1) negatively modulates multiple cellular pathways. These findings indicate that exosomal miR-1231 may act as a suppressor of pancreatic cancer aggressiveness (143). Zhou et al. developed a targeted exosomal delivery system, engineering extracellular vesicles (designated IEVs-PFD/138) co-loaded with miR-138-5p and the anti-fibrotic drug pirfenidone (PFD). These extracellular vesicles (EVs) were surface-modified with integrin α5-targeting peptides to facilitate precise delivery, enabling the reprogramming of cancer-associated fibroblasts (CAFs) and suppression of their tumor-promoting functions (102). In a parallel demonstration of exosome-mediated intercellular regulation, Qi et al. reported that miR-3173-5p, packaged within CAF-derived exosomes, was transferred to cancer cells upon uptake. This exosome-delivered miRNA subsequently functioned as a molecular sponge for ACSL4, thereby inhibiting ferroptosis in the recipient cancer cells (103). Despite these advances, each delivery platform presents distinct limitations in terms of biosafety, targeting efficiency, manufacturing scalability, and clinical applicability. Future research should concentrate on rational carrier design, combination delivery strategies, and rigorous preclinical and clinical validation to accelerate the translation of miRNA-based therapeutics for PDAC.

### 4.2 The EUS-FNI as the potential delivery system

Endoscopic ultrasound (EUS), introduced in the 1990s, has significantly revolutionized the diagnostic and therapeutic landscape of pancreatic tumors.Originally developed as a diagnostic modality, EUS has rapidly evolved into a versatile interventional platform. Recently, new therapeutic techniques, such as EUS-guided fine needle injection (EUS-FNI) or radiofrequency ablation (RFA), have been introduced for administering antitumor agents. Several intrinsic features make EUS particularly suitable for interventional therapy. The first and most crucial property is its high spatial resolution and the proximity of its transducer to the target lesion, allowing it to access small lesions while avoiding intervening structures, blood vessels, and air-filled organs. The second advantage lies in its minimal invasiveness and high safety profile in targeting pancreatic lesions. These have advanced this modality over interventional radiology and surgery in diverse pancreatic tumor treatment applications (144). EUS-guided FNI has been successfully adapted for a range of therapeutic interventions, including chemotherapy, immunotherapy, gene therapy, and intra-tumoral implantation (145–147). Levy et al. administered gemcitabine (38 mg/mL) via EUS-FNI in patients with PC before conventional therapy (148). The procedure was well tolerated, with no initial or delayed adverse events reported, and yielded a median overall survival of 10.4 months, compared to 6 months in metastatic pancreatic cancer patients receiving GEM alone. Similarly, direct administration of immunotherapeutic agents via EUS-FNI has been utilized for PDAC treatment. Irisawa et al. injected DCs under EUS guidance into patients with unresectable, gemcitabine-refractory pancreatic cancer (149). Among seven treated patients, five also underwent prior radiation therapy to induce apoptosis and facilitate tumor antigen cross-presentation. The procedure demonstrated excellent safety and tolerability, with no procedural adverse events or systemic toxicity. Four patients achieved stable disease for more than 6 months despite resistance to gemcitabine. In another study, the combination of gemcitabine and OK432-pulsed DCs administered through EUS-FNI effectively induced a T helper 1-type immune response, suggesting synergistic immunomodulatory potential (150).

Collectively, these findings underscore the promise of EUS-FNI as a safe and effective strategy for localized therapeutic delivery. Its ability to achieve targeted intratumoral administration with minimal systemic exposure makes it particularly attractive for future applications involving miRNA-based immunotherapies in PDAC.

### 4.3 Implantable devices as an alternative to nanoparticles

While direct intratumoral injection provides a localized treatment approach, its utility is often constrained by rapid agent dissipation and heterogeneous intratumoral distribution, which can compromise efficacy and lead to unintended systemic exposure. To overcome these limitations, the implantable devices, including scaffold-assisted platforms and nanofluidic drug-eluting seed (NDES), have been developed to facilitate a controlled release of therapeutic agents at specific tissue sites (151). Lee et al. introduced a deeply implantable, wirelessly operated, and shape morphing, 3D micro-LED (SMLED) for minimally invasive photodynamic therapy (mPDT) in the intra-body environment (152). The ultra-thin (15 µm) SMLED features a windmill-shape architecture, allowing it to conform to complex 3D curved surfaces of tumor tissues. The self-shape morphing characteristics of SMLED, in response to fluids and temperature, allow for precise, selective PDT, minimizing the risk of unwanted apoptosis/necrosis due to detachment from the volumetrically changing PDAC during treatment. A wireless power transfer (WPT) system for *in vivo* mPDT is established by integrating SMLED with circuits and inductive coils. Scaffold-assisted drug delivery systems have been used to enhance cancer immunotherapies through locally delivering immunotherapeutic agents such as antibodies, cytokines, and CAR-T cells. Particularly, biomaterial scaffolds such as hydrogels, microneedles, biopolymer implants, and cryogels open new horizons for assisting local anticancer treatment by precisely delivering immunotherapeutic agents and recruiting immune cells to the scaffold site (153). In a study by Zhan et al., a drug-eluting scaffold loaded with CCL17 was fabricated via electrospinning of polyglyconate and porcine gelatin (154). This scaffold successfully recruited murine CCR4^+^CD8^+^ T cells into PDAC, suppressed hepatic metastasis, and showed no cytotoxicity *in vitro*. PDAC remains challenging to treat due to its dense stromal barrier, which limits drug penetration during systemic therapy. Traditional systemic therapies are ineffective at penetrating the dense tumor stroma. A recent study demonstrated that continuous low-dose intratumoral administration of CD40 mAb using the nanofluidic drug-eluting seed (NDES) can alter the tumor microenvironment to decrease tumor burden in mouse models. NDES significantly reduced tumors at a quarter of the systemic treatment dosage without causing related adverse events (155). Collectively, these studies suggest great potential for drug delivery for immunotherapy in PDAC. However, further investigation is needed to address clinical considerations. Device-tumor size matching, biocompatibility, and safety require careful optimization. In addition, release kinetics must be precisely calibrated to align with the dynamic processes of the cancer immunity cycle. Additional preclinical and clinical research is needed to refine these implantable systems for safe and effective use in pancreatic cancer therapy.

In summary, efficient and precise delivery remains the pivotal determinant for the successful clinical translation of miRNA-based therapy in pancreatic cancer. Emerging platforms, including advanced nanoparticles, engineered exosomes, EUS-guided intratumoral injection, and implantable drug-eluting devices, are progressively overcoming biological barriers such as dense stroma and limited drug penetration. While each system still faces challenges in safety, targeting accuracy, and scalability, continuous innovation in biomaterials and interventional technologies is rapidly reshaping the therapeutic landscape. Future efforts integrating optimized carriers with personalized miRNA selection are expected to enhance efficacy and accelerate the transition of miRNA-based strategies from experimental models to routine clinical application.

## 5 Discussion

This review synthesizes current evidence on the landscape of immunotherapy in PDAC and underscores the emerging role of miRNAs in modulating therapeutic response. Despite considerable advances in immune checkpoint inhibition and adoptive cell therapies, PDAC remains largely refractory to conventional immunotherapies, largely attributable to its complex and immunosuppressive TME. We have delineated how miRNAs function as pivotal regulators of immune homeostasis, influencing not only immune checkpoint molecules such as PD-L1 but also the activity of CAR-T cells, NK cells, and various stromal components within the TME.

Distinct from earlier reviews, this work extends beyond summarizing the regulatory roles of miRNAs in immune modulation and TME remodeling to also examine potential miRNA-based immunotherapy strategies. To improve the precise target of miRNAs, we introduce traditional delivery systems and further emphasize a forward-looking convergence of cutting-edge technologies, including EUS-FNI, miniature soft robotics, and advanced 3D imaging for the design and implementation of next-generation miRNA delivery systems. By integrating recent insights into miRNA-mediated immunoregulation with engineered and digital innovations, this review proposes a convergent roadmap toward more effective, precisely tailored, and patient-centric immunotherapeutic strategies for PDAC.

While miRNAs exhibit great advantages in their multi-targeted capacity, low drug resistance and dose safety, the clinical translation of miRNAs failed in several clinical trials. Hence, the translation of miRNA-based therapies into clinical practice for pancreatic cancer presents a multi-faceted challenge that spans target identification, delivery optimization, and clinical adaptation. A primary hurdle lies in selecting therapeutically relevant miRNAs from the vast pool of differentially expressed candidates. Given their pleiotropic nature, priority should be given to miRNAs capable of coordinately regulating multiple immunosuppressive pathways. This necessitates the integration of multi-omics data from large clinical cohorts, encompassing transcriptomic, proteomic, and metabolomic profiles to systematically uncover miRNAs that target central molecular networks in pancreatic cancer. Artificial intelligence (AI) and machine learning offer powerful computational tools to advance this process, as they can decode complex miRNA-mRNA regulatory networks, refine target prediction, and identify predictive biomarkers of immunotherapy response (156, 157). Nowadays, AI could be applied to identify significant mRNA and microRNA panels to classify the subtype in Renal cell carcinoma (RCC) (158). However, the integration of AI into clinical miRNA research remains in its early stages, which is currently limited to miRNA selection as potential biomarkers in PDAC, highlighting the need for dedicated development of AI-based pipelines for candidate selection and validation (159). In future, AI could be applied for miRNA-based therapy in PDAC via several aspects. First, AI could be applied to classify the subtype of PDAC via miRNA indicators. Second, when integrated with multi-omics data, AI could help identify and elucidate the targets and mechanisms of miRNA-based therapy. Last, AI might calculate the appropriate safe dosage for clinical trials that could mitigate the toxicity of miRNA-based therapy.

Beyond target selection, the clinical translation of miRNA-based therapeutics is constrained by several non-delivery-related barriers that require systematic mitigation strategies. These include biological challenges such as off-target effects, competition with endogenous miRNAs for RNA-induced silencing complex (RISC) loading, and potential disruption of native regulatory networks, which may attenuate therapeutic efficacy or destabilize native gene regulatory equilibria. These challenges highlight the importance of rational sequence design, including seed-region optimization and transcriptome-informed targeted filtering, to enhance specificity while preserving network robustness. Pharmacokinetic limitations further impede translation, as unprotected RNAs are rapidly degraded by nucleases, exhibit poor membrane permeability, and often undergo renal clearance or accumulate in non-target organs. While chemical stabilization and carrier-based protection can improve systemic exposure, they must be carefully balanced against altered biodistribution. Consequently, the development of chemically modified miRNAs with improved stability, combined with tissue-biased or ligand-guided delivery strategies, represents a key direction for achieving therapeutically relevant exposure at target sites. Toxicity, arising from sources such as on-target over-suppression, immune activation, or carrier-related effects, poses another major barrier. Addressing these risks requires a multi-layered mitigation strategy involving advanced chemical modifications, intelligent sequence design, low-immunogenicity carriers, and careful dose optimization based on the minimal effective dose principle, rather than maximal target inhibition. Moreover, the translational gap between preclinical models and human disease is driven by species-specific differences in miRNA expression, immune interactions, disease heterogeneity. Bridging this gap will require more physiologically relevant experimental systems, such as humanized models, patient-derived organoids, and biomarker-guided translational studies, to ensure the miRNA modulation observed preclinically is predictive of clinical benefit.

**Figure 6:**
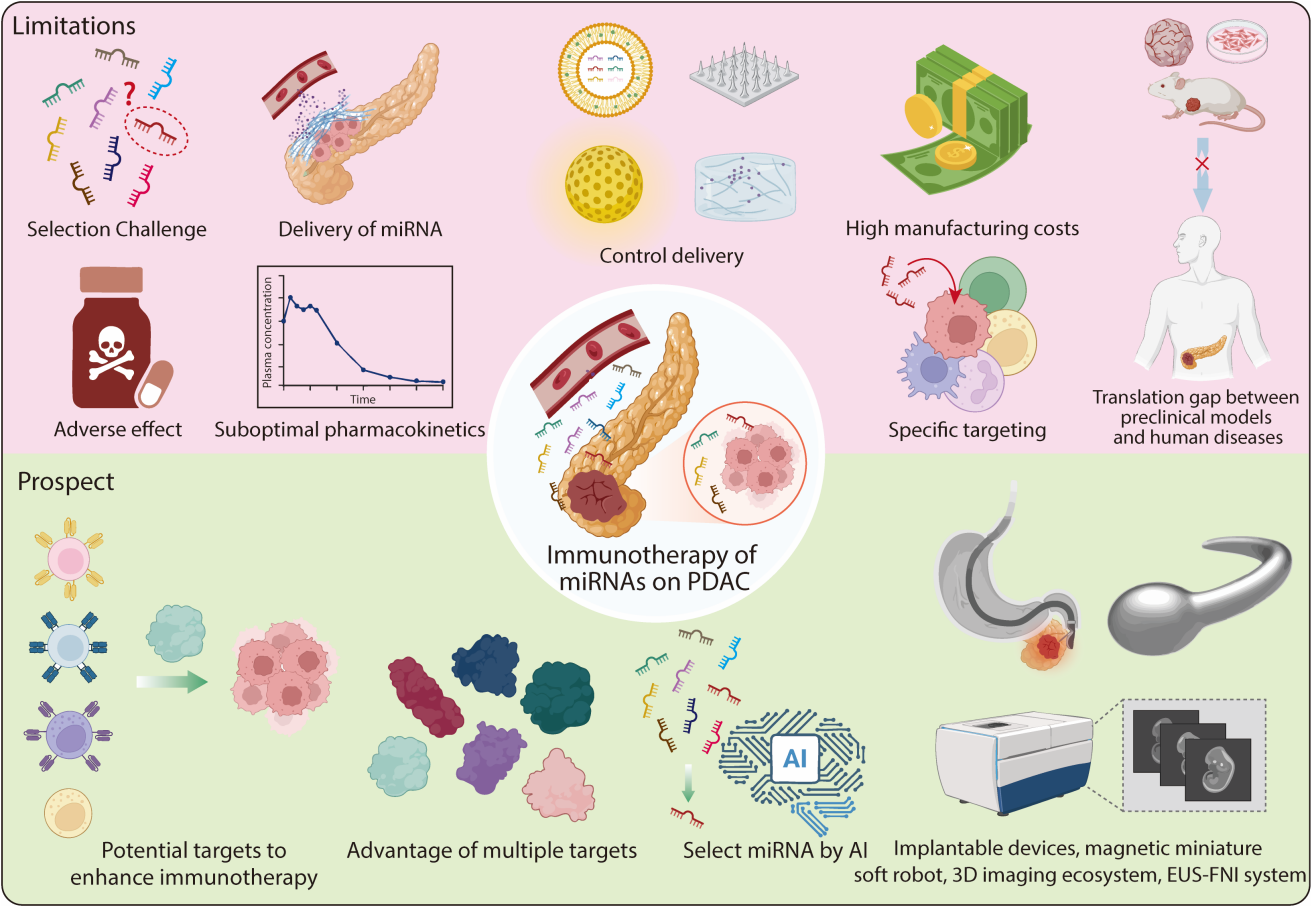
The limitations and prospect for immunotherapy of microRNAs on PDAC. The limitations of miRNA-based immunotherapy for PDAC include the following points: First, it is difficult to identify the most efficient miRNA for immunotherapy in PDAC. Second, adverse effects. Third, delivery of miRNA. Fourth, controlling the delivery of miRNA. Fifth, achieving precise targeting of specific cells or places. Sixth, translation gap between preclinical models and human diseases. Seventh, suboptimal pharmacokinetics of miRNAs. Eighth, high manufacturing costs of miRNA. The prospect of miRNA-based immunotherapy for PDAC includes: First, potential targets to enhance the efficiency of immunotherapy in PDAC. Second, miRNA can target multiple molecules to achieve regulation of multiple molecules and pathways. Third, the application of technologies like implantable devices, EUS-FNI system, magnetic miniature soft robots and more precise 3D imaging ecosystems to improve immunotherapy. Fourth, selecting miRNAs using AI to improve efficiency. EUS-FNI, endoscopic ultrasound-guided fine needle injection; AI, artificial intelligence.

Delivery remains a central obstacle, as current systems, including viral vectors, lipid nanoparticles, and exosome-based carriers, struggle with stability, immunogenicity, inadequate tumor targeting, and limited penetration through the dense pancreatic stroma. These limitations underscore the need for delivery strategies that are both stroma-permissive and immunologically compatible. Although localized strategies such as implantable devices or EUS-guided intratumoral injection offer potential solutions, they face their own challenges in precise placement, biocompatibility, and manufacturability. Addressing these issues will likely require the integration of smart biomaterials with tunable degradation and release kinetics, as well as image-guided procedures to improve precision and consistency. Looking forward, emerging technologies such as magnetically guided soft microrobots and high-precision 3D imaging systems hold promise for enabling real-time navigation and spatially controlled delivery within the tumor microenvironment (160, 161). Although still at an early stage, these platforms conceptually enable active navigation through stromal barriers and localized payload release, potentially overcoming the diffusion-limited nature of conventional nanocarriers. By converging these advanced delivery platforms with rationally selected, network-modulating miRNAs, it may become feasible to achieve spatiotemporal remodeling of the tumor immune landscape, effectively converting immunologically cold pancreatic tumors into therapy-responsive lesions. Ultimately, the successful clinical translation of miRNA-based immunotherapy in pancreatic cancer will depend on an integrated, multidisciplinary effort to navigate these intertwined biological, pharmacological, and technological challenges. In this context, converting immunologically “cold” pancreatic tumors into therapy-responsive lesions may become achievable not through increased dosage, but through precise spatial and temporal control of miRNA activity (**Figure 6**).

Ultimately, the clinical translation of miRNA-based immunotherapy for pancreatic cancer will depend on an integrated, multidisciplinary strategy that aligns delivery engineering, tumor immunobiology, and translational feasibility, ensuring that technological innovation is matched with biological relevance and clinical practicality.

## 6 Conclusion

PDAC remains one of the most lethal malignancies of the upper gastrointestinal tract cancer. Although immunotherapy has revolutionized the treatment landscape of many solid tumors, its clinical efficacy in PDAC has been limited by the highly immunosuppressive and desmoplastic “cold” TME. MiRNAs have emerged as promising modulators of both tumor immunity and the TME, representing potential therapeutic targets and adjuncts to existing immunotherapeutic strategies. However, the successful translation of miRNA-based immunotherapy in PDAC requires the development of precise, efficient, and tumor-specific delivery systems capable of overcoming the physical and immunological barriers that characterize this disease.

In this review, we have summarized the current advances in miRNA-mediated immunoregulation and drug delivery strategies in PDAC, providing new insights and conceptual frameworks for future therapeutic development. Despite ongoing challenges related to target selection, delivery optimization, and clinical validation, the integration of multi-omics profiling, AI-driven analytics, and innovative delivery technologies offers a comprehensive pathway to surmount these limitations.

Moving forward, interdisciplinary collaboration among oncologists, bioengineers, computational biologists, and material scientists will be essential to accelerate the translation of miRNA-guided immunotherapies from bench to bedside. Such collaborative efforts hold great promise to transform PDAC from an immune-refractory malignancy into one responsive to immunotherapy, ultimately improving outcomes for patients afflicted by this devastating disease.

## Glossary

PC: pancreatic cancer
TME: tumor microenvironment
AI: artificial intelligence
PDAC: pancreatic ductal adenocarcinoma
PanIN: pancreatic intraepithelial neoplasia
miRNA: microRNA
ICB: immune checkpoint blockade
ACT: adoptive cell therapy
ORRs: overall response rates
NK cells: natural killer cells
PBMCs: peripheral blood mononuclear cells
CAR-M: chimeric antigen receptor macrophages
GVHD: graft versus host diseases
CAR-M-c-MET: chimeric antigen receptor macrophages targeting c-MET
CTLs: cytotoxic T lymphocytes
CAFs: cancer-associated fibroblasts
nt: nucleotide
tsmiR: tumor suppress microRNA
AMO: anti-miR oligonucleotides
LNA: locked nucleic acid
CTLA-4: cytotoxic T lymphocyte-associated protein-4
RCC: renal cell carcinoma
CRS: cytokine release syndrome
NKG2DLs: NKG2D ligands
MICA: major histocompatibility complex class I chain A
im-miRNAs: immune-modulatory miRNAs
PD-L1: programmed death ligand-1
FAK: focal adhesion kinase
IL-10: interleukin-10
TGF-β: transforming growth factor-β
VEGF: vascular endothelial growth factor
PFD: pirfenidone
PTC: papillary thyroid carcinoma
MDSCs: myeloid-derived suppressor cells
TAMs: tumor-associated macrophages
DCs: dendritic cells
NKT: natural killer T cells
BMI1: B-cell-specific Moloney murine leukemia virus insertion site 1
ADs: adenoviruses
AAVs: adeno-associated viruses
RVs: retroviruses
GEM: gemcitabine
NP: nanoparticle
LNPs: lipid nanoparticles
OMe-PS: O-methyl phosphorothioate
exomiR-34a: miR-34a coated with exosomes
EUS: endoscopic ultrasound
EUS-FNI: EUS-guided fine needle injection
RFA: radiofrequency ablation
NDES: nanofluidic drug-eluting seed
SMLED: 3D micro-LED
mPDT: minimally invasive photodynamic therapy
WPT: wireless power transfer
PARP: Poly (ADP-ribose) polymerase
MMR: mismatch repair
HRR: homologous recombination repair
EMT: epithelial-mesenchymal transition
BER: base excision repair
PARPis: PARP inhibitors
SSB: single-strand break
DSBs: double-strand breaks
NHEJ: non-homologous end joining
VEGFR: vascular endothelial growth factor receptors
HDAC: histone deacetylases
BET: bromodomain and extraterminal domain
tsmiRs: tumor-suppressive miRNAs
oncomiRs: oncogenic miRNAs
EPR: enhanced permeability and retention
RISC: RNA-induced silencing complex
BM-MSC: bone marrow mesenchymal stem cell
G-CSF: granulocyte colony-stimulating factor
CRT: chemoradiotherapy
PFS: progression-free survival
LAPC: locally advanced pancreatic cancer
IMRT: intensity-modulated radiotherapy
SBRT: stereotactic body radiotherapy
SMART: stereotactic magnetic resonance-guided adaptive radiotherapy
TILs: tumor-infiltrating lymphocytes
SCALOP: Selective Chemoradiation in Advanced LOcalised Pancreatic Cancer
ABC: ATP-binding cassette
EVs: extracellular vesicles
ROS: reactive oxygen species
